# Identification and Expression of the CCAP Receptor in the Chagas’ Disease Vector, *Rhodnius prolixus*, and Its Involvement in Cardiac Control

**DOI:** 10.1371/journal.pone.0068897

**Published:** 2013-07-09

**Authors:** Dohee Lee, Jozef Vanden Broeck, Angela B. Lange

**Affiliations:** 1 Department of Biology, University of Toronto Mississauga, Mississauga, Ontario, Canada; 2 Animal Physiology and Neurobiology, Katholieke Universiteit Leuven, Leuven, Belgium; Max-Delbrück Center for Molecular Medicine (MDC), Germany

## Abstract

*Rhodnius prolixus* is the vector of Chagas’ disease, by virtue of transmitting the parasite *Trypanosoma cruzi.* There is no cure for Chagas’ disease and therefore controlling *R. prolixus* is currently the only method of prevention. Understanding the physiology of the disease vector is an important step in developing control measures. Crustacean cardioactive peptide (CCAP) is an important neuropeptide in insects because it has multiple physiological roles such as controlling heart rate and modulating ecdysis behaviour. In this study, we have cloned the cDNA sequence of the *CCAP* receptor (*RhoprCCAPR*) from 5^th^ instar *R. prolixus* and found it to be a G-protein coupled receptor (GPCR). The spatial expression pattern in 5^th^ instars reveals that the *RhoprCCAPR* transcript levels are high in the central nervous system, hindgut and female reproductive systems, and lower in the salivary glands, male reproductive tissues and a pool of tissues including the dorsal vessel, trachea, and fat body. Interestingly, the *RhoprCCAPR* expression is increased prior to ecdysis and decreased post-ecdysis. A functional receptor expression assay confirms that the RhoprCCAPR is activated by CCAP (EC_50_ = 12 nM) but not by adipokinetic hormone, corazonin or an extended FMRFamide. The involvement of CCAP in controlling heartbeat frequency was studied *in vivo* and *in vitro* by utilizing RNA interference. *In vivo,* the basal heartbeat frequency is decreased by 31% in bugs treated with dsCCAPR. Knocking down the receptor in dsCCAPR-treated bugs also resulted in loss of function of applied CCAP *in vitro.* This is the first report of a GPCR knock-down in *R. prolixus* and the first report showing that a reduction in *CCAPR* transcript levels leads to a reduction in cardiac output in any insect.

## Introduction

Neuropeptides and their receptors are vital components in insects since they regulate physiological and behavioral processes associated with development, reproduction and metabolism. Targeting the ligand-receptor interactions is an important strategy for developing therapeutics/pest control agents in the pharmaceutical and agrochemical industries [1]–[6]. These might be used as potential agonist or antagonist of the receptor by interfering with normal functioning within the animal. One such insect for which control is required is the blood-feeding bug, *Rhodnius prolixus*, which is the vector of Chagas’ disease in Central and South America.

Crustacean cardioactive peptide (CCAP) is a cyclic nonapeptide that is conserved in arthropods. CCAP was first isolated as a cardioaccelerator from the pericardial organs of the shore crab, *Carcinus maenas* [7], [8]. Later, it was biochemically isolated from the nervous system of insects [9], [10]. The important functions of CCAP in insects have been well documented. For example, CCAP triggers increasing heart rate in *Manduca sexta* and *Drosophila melanogaster* [11], [12], controls and regulates ecdysis behavior by modifying central motor programs in *M sexta* and *D. melanogaster* [13], modulates oviduct contractions in *Locusta migratoria* [14], [15], and regulates enzyme secretion in *Periplaneta americana* [10].

The receptor for CCAP is a G-protein coupled receptor (GPCR). G-protein coupled receptors are important signal transducing receptors since they mediate the effects of many neuropeptides [2], [16]–[18]. GPCRs typically contain 7 alpha-helical transmembrane segments. They can transduce extracellular signals into cellular physiological responses through the activation of a heterotrimeric G protein [2], [16]–[18]. CCAP receptors have been cloned in several insect species [19]–[21]. In *D. melanogaster*, the CCAP receptor is expressed in all developmental stages, with the highest expression in adult heads [19]. Recently, CCAP receptor expression was observed in all developmental stages of the mosquito with a peak in second instar larvae and pupae [22]. In *Tribolium castaneum*, two CCAP receptors have been isolated, CCAPR1 and CCAPR2, and only CCAPR2 is essential for eclosion behaviour [21].

In the current study, we have isolated the cDNA sequence of the CCAP receptor in the medically-important blood-gorging bug, *Rhodnius prolixus.* The *R. prolixus* CCAP receptor (*RhoprCCAPR*) was cloned from a CNS cDNA library and in a functional assay shown to be activated by CCAP with an EC_50_ value of 12.2±1.1 nM. Quantitative real-time PCR (qPCR) analysis revealed spatial expression patterns of *RhoprCCAPR* in the CNS as well as peripheral tissues of 5^th^ instar *R*. *prolixus*. The *RhoprCCAPR* transcript level increased prior to ecdysis and decreased post-ecdysis. We have also confirmed the cardioacceleratory functions of CCAP in the adult male *R. prolixus* and demonstrated that its cardioacceleratory effect is abolished when the *RhoprCCAPR* transcript is knocked down by RNA interference (RNAi).

## Materials and Methods

### Animals

The *R. prolixus* was obtained from a colony that is fed once during each instar with defibrinated rabbit blood (Hemostat Laboratories, Dixon, CA, USA; supplied by Cedarlane, Burlington, ON, Canada). Gorging is the stimulus for growth and development to the next instar. The instars used in these experiments were allowed to gorge and then held in an incubator at 30% humidity and 28°C in a 16 h:8 h light/dark cycle.

### Isolation of the *RhoprCCAPR* Transcript from 5th Instar *R. prolixus*

The sequence of RhoprCCAP receptor was aligned with the corresponding *D. melanogaster* (AAO66429), *Aedes aegypti*, (XP_001659389), *T. castaneum* (ABN79651, ABN79652), and *Apis mellifera* (XP_001122652) receptors using Clustal W [23]. Conserved regions of amino acid sequences were used in a TBLASTN search against the complete *R. prolixus* genome database. The contig was constructed and the putative CCAP receptor encoding the nucleotide sequence was obtained. Based on the predicted CCAP receptor sequences, gene specific primers (GSP) were designed (Table 1A). The procedure of cloning the *CCAPR* cDNA sequence has been performed as previously described [24]. All PCR conditions were identical: 5 min at 95°C, 30 sec at 94°C, 30 sec at 57°C, 60 sec at 72°C and 10 min at 72°C.

**Table 1:** Gene-specific primers (GSP) for the CCAP receptor in *R. prolixus*.

A) Oligo name	Oligo Sequence (5′ to 3′)
**5′ RACE primers**	
CCAPR_FOR1	AGCACTGGATAATGGACTGG
CCAPR_FOR2	AGCATTTGCAGATTTATCAGTTG
CCAPR_FOR3	ATCGTCTGGATGCAATTACAAG
CCAPR_FOR4	GGTGGAGATAAGGGAGATGAC
CCAPR_FOR5	CCACGTTTATTCAAAGTCTTGC
**3′ RACE primers**	
CCAPR_REV1	GATTATCTTGGTTACGAATAGTGG
CCAPR_REV2	GGC TAC TGC GAT ATT TGT TTG AG
CCAPR_REV3	TGT CAT CTC CCT TAT CTC CAC C
CCAPR_REV4	TCCATTCAACCGTGATCC
CCAPR_REV5	CCC AAA ACA ATC GCT GC
**B) Oligo name**	**Oligo Sequence (5′ to 3′)**
**CCAPR specific primer for qPCR**	
qPCR _CCAPR_F1	GCTTAGCACTGGATAATGGACTG
qPCR _CCAPR_R1	TCAATACGCTGATCAGTCCAACT
**Reference genes for qPCR**	
qPCR _Actin F1	AGAGAAAAGATGACGCAGATAATGT
qPCR _Actin R1	CGGCCAAATCCAATCG
qPCR _RP48_F1	GTGAAACTCAGGAGAAATTGGC
qPCR _RP48_R1	GCATCATCAACATCTCTAATTCCTTG
**C) Oligo name**	**Oligo Sequence (5′ to 3′)**
**Primers to amplify full ORF**	
CCAPR_ORF_F1	TTAGCACTGGATAATGGACTGG
CCAPR_ORF_F2	ATGGACTGGGTTATAAGAGATAATTAC
CCAPR_ORF_R1	CTATTCGTAACCAAGATAATCTCTAAATG
CCAPR_ORF_R2	CACTATTCGTAACCAAGATAATCTCTAA
**Primers for introduce Kozak sequence**	
Kozak_CCAPR_ORF_F2	GCCACC ATGGACTGGGTTATAAGAG

(A) Primers used for 5′ and 3′ rapid amplification of cDNA ends (RACE). (B) Transcript specific primers for real time PCR for the spatial expression profile. (C) Primers for cloning the complete open reading frame (ORF), and for introducing a Kozak translation initiation sequence at the 5′UTR.

### Sequences Analysis

The amino acid sequence of RhoprCCAPR and that of other insect CCAP receptors (*Anopheles gambiae* AnogaCCAPR1, AGAP001961; AnogaCCAPR2, XP_321100.4; *A. aegypti* AedaeCCAPR1, XP_001659389.1;AedaeCCAPR2, XP_001659388.1; *Culex pipiens* CulpiCCAPR1, CPIJ006268; CulpiCCAPR2, XP_001847670.1; *Culex quinquefasciatus* CulquCCAPR, XP_001847670; *D. melanogaster* DromeCCAPR, AAO66429.1; *D. virilis* DroviCCAPR, GJ23325; *D. mojavensis* DromoCCAPR, GI22912; *T. castaneum* TricaCCAPR1, ABN79651; TricaCCAPR2, ABN79652; *A. florea* ApiflCCAPR, Predicted XP_003691184; *A. mellifera* ApimeCCAPR, XP_001122652.2; *Bombyx impatiens* CCAPR, Predicted XP_003494126; *Megachile rotundata* MegroCCAPR, Predicted XP_003700512; *Nasonia vitripennis* NasviCCAPR, XP_001602277.1; *Acyrthosiphon pisum* AcypiCCAPR, Predicted XP_003245097, *B. mori* BommoCCAPR1, NP_001127724.1; BommoCCAPR2, NP_001127746.1) were aligned using Clustal W [23]. Also, the amino acid sequence of RhoprCCAPR was aligned with that of the putative AKH receptor in *A. pisum, D. melanogaster* and *B. mori*, as well as the putative corazonin receptor in *R. prolixus*. The aligned arthropod sequences which were either identical or similar to the consensus sequence, were colored with black or gray, respectively, by using the BOXSHADE 3.21 server (http://www.ch.embnet.org/software/BOX form.html). Phylogenetic analysis of the aligned sequences was produced using Molecular Evolutionary Genetics Analysis (MEGA) (version 4.0.4) [25].

### Preparation of the RhoprCCAPR Construct

The coding region of RhoprCCAPR (1,128 bp) (*RhoprCCAPR*) was amplified from unfed 5^th^ instar CNS cDNA with gene specific primers (Table 1C) using a Q5 High fidelity DNA polymerase (New England Biolabs, Pickering, ON). At the 5′ end before the ATG initiation codon, the Kozak translation initiation sequence (3′ ACCATG-5′) was introduced since this is required for optimal translation by eukaryotic ribosomes (Table 1C) [26]–[28]. The PCR product was run on a 0.8% agarose gel for 40 mins (160V) and the correct size of band was excised and purified (Promega, Madison, WI, USA). This was then subcloned into the pGEM T easy vector (Promega, Madison, WI) to verify its sequence. The insert with the Kozak sequence was excised using restriction enzyme (NotI) and subcloned into the expression vector, pcDNA™3.1(+) (Invitrogen, Carlsbad, CA) to express in Chinese hamster ovary (CHO-K1) WTA11, mammalian cells (Euroscreen S. A., Belgium; provided by Prof. Dr. M. Parmentier and Dr. M. Detheux; Brussels, Belgium).

### Functional Analysis of CCAP Receptor

*RhoprCCAPR* was transiently expressed in CHO-WTA11 cells that stably express promiscuous G_α16_ and apoaequorin. This cell line has been used extensively for insect GPCR functional assays [21]. The transfection was performed using X-treme HP DNA transfection reagent (Roche Applied Science, Indianapolis, IN) with the ratio of 3 (transfection reagent) to 1 (*RhoprCCAPR*/pcDNA™3.1(+) or empty pcDNA™3.1(+) vector) according to the manufacture’s protocol (Roche Applied Science, Indianapolis, IN). Cells were grown in the complete Dulbecco’s Modified Eagle Medium Nutrient Mixture F12-Ham (DMEM/F12, 10% fetal bovine serum, FBA, 100 IU/ml penicillin/streptomycin (Invitrogen, Carlsbad, CA)) in the 5% CO_2_ 37°C incubator. After 48 hours, transfected cells were incubated with 5 µM Coelentarazine for 4 hours (Invitrogen, Carlsbad, CA) at room temperature in the dark. Interaction between ligand and the cloned *RhoprCCAPR* leads to a bioluminescent response that is mediated by the IP_3_/Ca^2+^ signaling pathway. The luminescence assay was performed on opaque 96-well microplates (Corning, Lowell, MA). 50 µl of cells were introduced into each well of the 96-well plate that contained either controls or different concentration of peptides (RhoprCCAP, RhoprCorazonin, RhoprAKH and GNDNFMRFa) (Table 2) and the changes of luminescence were recorded for 20 sec. Wells only containing DMEM/0.1% BSA medium served as a negative control, while wells containing 50nM ATP served as a positive control. All luminescence values were corrected for background values from wells containing only DMEM/BSA medium and all values were calculated as maximum percentage difference. The response for each ligand concentration in replica wells and from at least three replica plates was averaged for analysis. GraphPad prism 5 (version 5.03) was used to analyze and generate the data.

**Table 2:** Summary of peptides tested on the CCAP functional receptor expression assay.

Peptide Name	Sequence	EC_50_ (M) RhoprCCAPR
RhoprCCAP	PFCNAFTGC-NH_2_	12.2×10^−9^
RhoprAKH	pQLTFSTDW-NH_2_	Not active
Corazonin	pQTFQYSRGWTN-NH_2_	Not active
ExtendedFMRFa	GNDNFMRF-NH_2_	Not active

### cDNA Synthesis from Various Tissues

CNS (brain, sub-oesophageal ganglion, prothoracic ganglion, mesothoracic ganglionic mass and stretches of abdominal nerves) and peripheral tissues (salivary glands, foregut, anterior midgut, posterior midgut, hindgut, Malpighian tubules, pool of tissues including dorsal vessel/trachea/fat body, female reproductive tissue and male reproductive tissue) were extracted from 4^th^, 5^th^ instar or male adult *R. prolixus* in physiological saline (NaCl, 150 mmol L^−1^, KCl, 8.6 mmol L^−1^, CaCl_2_, 2 mmol L^−1^, Glucose, 34 mmol L^−1^, NaHCO_3_, 4 mmol L^−1^, MgCl_2_, 8.5 mmol L^−1^, HEPES, 5 mmol L^−1^, pH 7.0) and stored in RNA later solution (Ambion, Austin, TX). Total RNA was isolated from tissues using the Trizol® reagent (Ambion, Austin, TX) according to the manufacturer’s protocols and quantified using a Nanodrop UV spectrophotometer (Thermo Scientific, Wilmington, Delaware, USA). 200 ng of total RNA from each tissue was used to synthesize cDNA using iScript™ Select cDNA Synthesis Kit (Bio-Rad, Mississauga, ON) according to the manufacturer’s protocols. CNS cDNA was diluted 20-fold using nuclease-free water and subsequently used as template for quantitative PCR (qPCR).

### Real Time PCR of RhoprCCAP Receptor

Real time PCR analyses were carried out on a CFX96Touch™ Real-Time PCR Detection System (Bio-Rad Laboratories Inc., Hercules, CA, USA) using the Ssofast™ EvaGreen supermix (Bio-Rad Laboratories Inc., Hercules, CA, USA). CNS and peripheral tissues from 5^th^ instar *R. prolixus* were dissected in physiological saline and stored in RNA later solution (Ambion, Austin, TX). Primers for *RhoprCCAPR* and reference genes (ribosomal protein 49, rp49 and actin 5c) (Table 1B) were designed to amplify target fragments of similar size across all samples [29]. Each primer set was designed with one primer over an exon/exon boundary and the primer efficiency was determined for each target. The amplification conditions were as follows: initial denaturation at 95°C for 30 sec, 40 cycles of denaturation at 95°C for 5 sec, annealing at 60°C for 5 sec, and extension at 72°C for 5 sec. The melting curve analysis was performed and all qPCR products were run on a 1% agarose gel. The relative expression was determined following the ΔΔCt method [30], [31] and fold-differences were normalized to both reference genes, RP49 and actin 5c. qPCRs were repeated for a total of three biological replicates with three technical replicates each that included a no template control and a no reverse-transcriptase control.

### Double-stranded RNA Synthesis

*RhoprCCAPR* transcripts were amplified using PCR from CNS cDNA library. As a control, the ampicillin resistance (*ARG*) gene was PCR amplified from the pGEM-T Easy Vector system (Promega, Madison, WI, USA). Then, 1 µl of the PCR product was amplified by gene specific PCR primers (Table 3) that were conjugated with 23 bases of the T7 RNA polymerase promoter at the 5′ end (5′-taatacgactcactatagggaga-3′) (Table 3). All PCR amplification conditions were as follows: 5min initial denaturation for 5mins at 94°C, 35 cycles for 30 sec at 94°C, for 30 sec at 58°C, for 60 sec at 72°C, and final extension for 10 min at 72°C. The PCR products were used as a template for double stranded RNA (dsRNA) synthesis using the T7 Ribomax Express RNAi System (Promega, Madison, WI, USA). After synthesis, the dsRNA was precipitated with isopropanol, eluted in DEPC treated water, and then quantified at 260_ nm_ wavelength using nanodrop. The quality of the dsRNA products was verified by 1% agarose gel electrophoresis and kept at −80°C until use. Before injection, the dsRNA was resuspended with DEPC treated water in the 2 µg/µl.

**Table 3:** Primers used to generate the double strand RNA (dsRNA) of the RhoprCCAPR and the ampicillin resistance gene (ARG).

RNAi constructs	Oligo Sequence (5′ to 3′)
**Primers to amplify RhoprCCAPR**	
dsCCAPR_For1	CTGGATAATGGACTGGGTTATAAG
dsCCAPR_For2	TATCTGGAGGATCACGGTTG
dsCCAPR_Rev1	GAATAGTGGCTCTGCGTAACG
dsCCAPR_Rev2	TACTGGATTAGCTGCTGAATTGAG
**Primers to amplify ARG**	
dsARG_FOR1	ATGAGTATTCAACATTTCCGTGTC
dsARG_FOR2	CAACAGCGGTAAGATCCTTG
dsARG_REV1	GGCACCTATCTCAGCGATC
dsARG_REV2	AATAGTTTGCGCAACGTTG
**Primers to generated dsRhoprCCAPR**	
T7_dsCCAPR_For1	TAATACGACTCACTATAGGGAGACTGGATAATGGACTGGGTTATAAG
T7_dsCCAPR_For2	TAATACGACTCACTATAGGGAGATATCTGGAGGATCACGGTTG
T7_dsCCAPR_Rev1	TAATACGACTCACTATAGGGAGAGAATAGTGGCTCTGCGTAACG
T7_dsCCAPR_Rev2	TAATACGACTCACTATAGGGAGATACTGGATTAGCTGCTGAATTGAG
**Primers to generated dsARG**	
T7_dsARG_FOR1	TAATACGACTCACTATAGGGAGAATGAGTATTCAACATTTCCGTGTC
T7_dsARG_FOR2	TAATACGACTCACTATAGGGAGACAACAGCGGTAAGATCCTTG
T7_dsARG_REV1	TAATACGACTCACTATAGGGAGAGGCACCTATCTCAGCGATC
T7_dsARG_REV2	TAATACGACTCACTATAGGGAGAAATAGTTTGCGCAACGTTG

The T7 promoter site is denoted as bold in the sequence of dsCCAPR and dsARG.

### dsRNA Delivery

Adult *R. prolixus* were anesthetized with CO_2_ for 10 sec and 1 µl of 2 µg of dsCCAPR was injected into the thorax using a 5 µl Hamilton syringe to knock down *RhoprCCAPR* transcript levels. As a control, groups of *R. prolixus* were injected with either 1 µl of dsARG or had no injection. *R. prolixus* were left for 1 hour at room temperature to recover and then placed into an incubator at 28°C on a 16 h:8 h light/dark cycle.

### Verification of dsRNA Knockdown Using Real Time PCR

Four CNS or peripheral tissues (pool of tissue containing fat body, trachea and dorsal vessel) were collected from adults that had been injected with dsARG, dsCCAPR as well as no treatment. Total RNA was extracted using the Trizol® reagent (Life Technologies Corporation, Carlsbad, CA, USA) and the cDNA was synthesized using iScript™ Select cDNA Synthesis Kit (Bio-Rad, Mississauga, ON). To verify the efficiency of synthesized dsRNA, qPCR were performed as described above.

### Heartbeat Assay

#### Visual detection of *in vivo* heartbeat

In this experiment, the heartbeat rate of adult males that had been injected with 2 µg of dsRNA (either dsCCAPR or dsARG) was measured 2 days after injection. Day 2 was chosen since preliminary experiments indicated that greater than 80% knockdown was obtained by this time and this efficiency was similar for days 3 to 5. Animals were immobilized ventral-side down on a Dental wax-coated dissecting dish and their wings were spread gently and held in place by the wax. The heartbeat was observed under a dissecting microscope with a 10× objective focused on the heart through the relatively transparent dorsal cuticle. Each bug was left for 5 minutes under the light of the dissecting microscope and then the heartbeat was counted per minute.

#### *In vitro* heartbeat

*In vitro* heartbeat was monitored as described in Lee and Lange (2011) with minor modifications. The ventral cuticle, digestive and reproductive systems were dissected and removed under RNase free saline. The dorsal vessel and dorsal diaphragm remained attached to the dorsal cuticle. Then, the dorsal cuticle was placed onto a Sylgard-coated dissecting dish and covered with 100 µL of saline. Electrodes attached to an impedance converter (UFI model 2991, Morro Bay, CA, USA) were placed between the fifth and sixth abdominal segments on either side of the dorsal vessel anterior to the alary. The preparation was stabilized in 100 µL of saline for 10 min at room temperature, and then 50 µL of 10^−9^M CCAP was exchanged for 50 µL of saline. Heartbeat frequency was measured from the traces observed on a Linear Flat-bed single channel chart recorder. The preparation was washed with saline for 5 mins post application of CCAP. Heartbeat frequency was determined for 1 min before and after the application of 10^−9^M CCAP. The response to 10^−9^M CCAP was quantified by measuring the increase in frequency compared to saline, which was then expressed as a percentage of the maximum change in frequency for each preparation. Pools of tissues including dorsal vessel/fat body/trachea were collected and the percentage of knockdown was measured by qPCR as indicated above.

## 3.0. Results

### 

#### Rhodnius prolixus CCAP receptor

**Figure 1:**
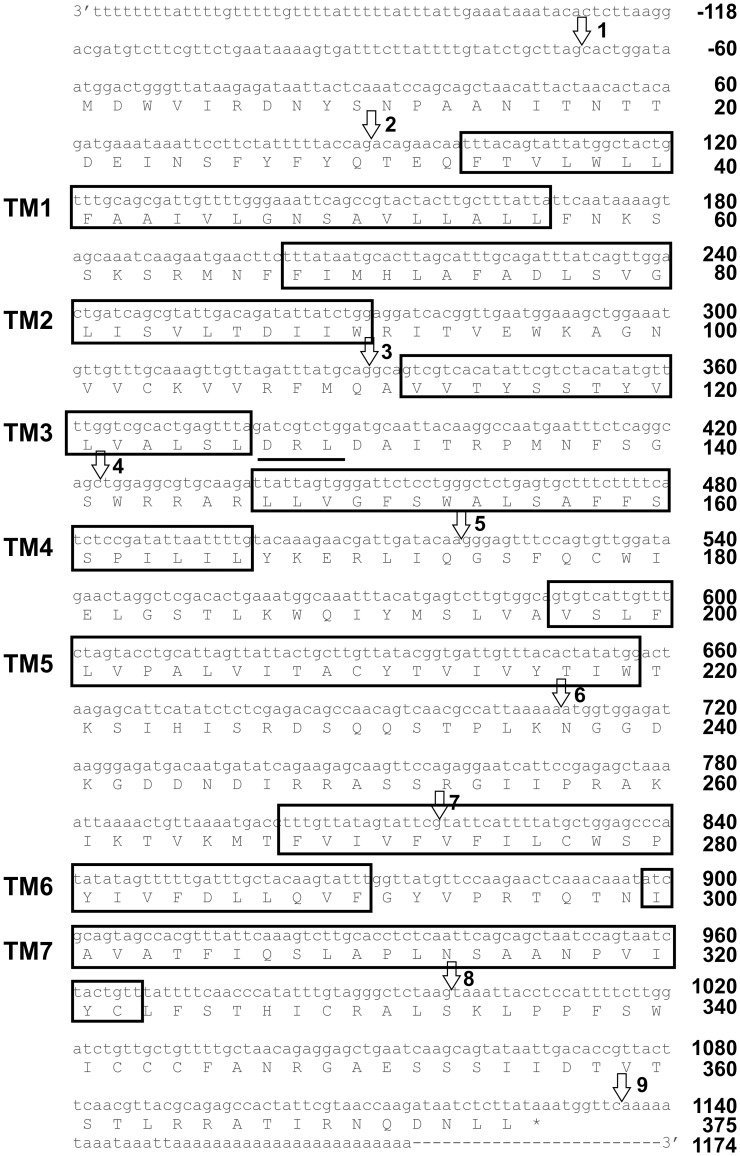
cDNA sequence of the *RhoprCCAP* receptor and deduced translation in 5^th^ instar *R. prolixus*. A) Nucleotide and amino acid sequences of the coding region starts at the nucleotide sequence ATG. Asterisk refers to the stop codon (TGA). The predicted transmembrane domains are box outlined and numbers on the left margin denotes the predicted transmembrane domains (TM1-7). The modified GPCR DRL sequences at amino acid residue 127 to 129 are underlined. The locations of introns (intron 1 to 9) are indicated by arrows.

**Figure 2:**
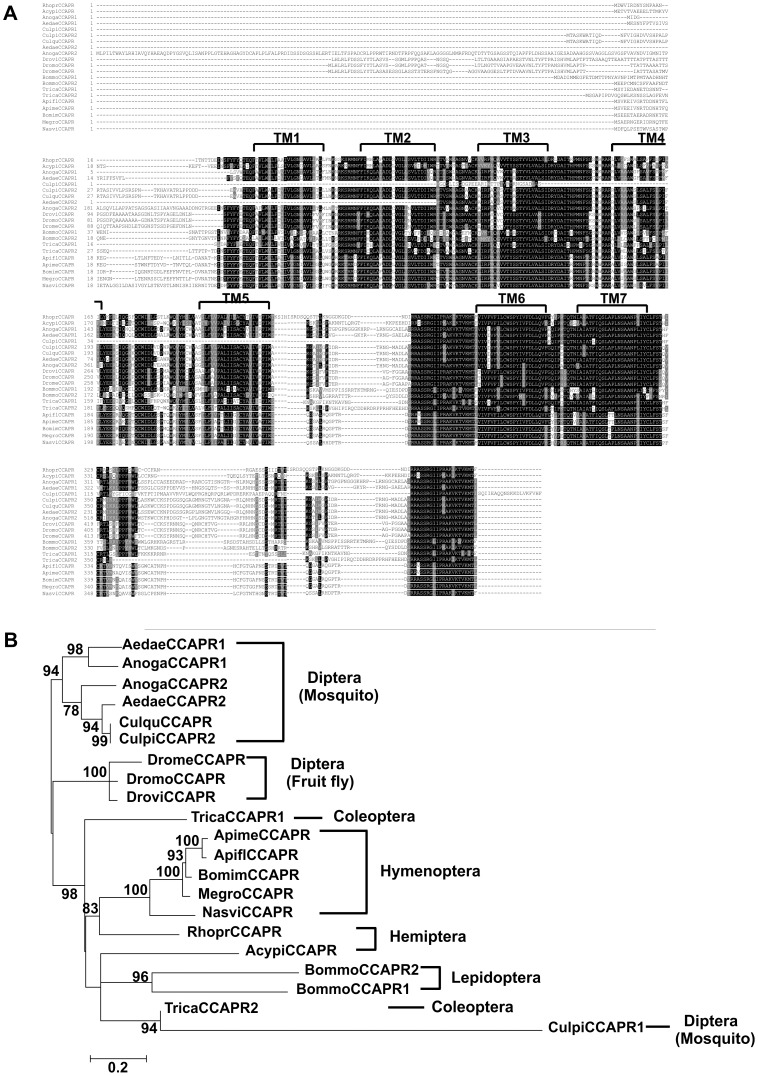
Protein alignment and phylogenetic analysis of CCAP receptors in insects. A) Amino acid sequence alignment of the CCAP receptors identified or predicted in 21 species in arthropods. The predicted location of the seven transmembrane regions (TM1-TM7) are indicated above each row. Following the 50% majority rule, identical amino acids are shaded in black, and similar amino acids are shaded in gray in column consensus residues. (B) The phylogenetic relationship of the insect CCAP receptors was generated using the Maximum Likelihood method based on the Jones et al. (1992) with frequency model [45]. The numbers at the nodes represent percentage support in 1500 bootstrap replicates. All positions containing gaps and missing data were eliminated. This phylogenetic tree is drawn to scale and the branch lengths are measured in the number of substitutions per site.

The *RhoprCCAPR* (Accession number: KC004225) was cloned from a 5^th^ instar cDNA CNS library [see 32] using a modified rapid amplification of cDNA ends (RACE) [32]. The *RhoprCCAPR* sequence consists of 1279 nucleotides, which code for a polypeptide of 374 amino acid residues (Figure 1). The *RhoprCCAPR* has 10 exons that are separated by 9 introns and is predicted to have 7 alpha-helical transmembrane segments (TM) in the open reading frame (ORF) with three extra- and three intracellular loops as well as an intracellular C-terminal tail using TMHMM server, v.2.0 (http://www.cbs.dtu.dk/services/TMHMM/). The sequence analysis of the RhoprCCAPR revealed the characteristics of a rhodopsin-like GPCR [29]. It also showed the presence of a divergent DRL sequence at amino acid residue 127 to 129, instead of the DRY sequence motif that can be found in many GPCRs belonging to the rhodopsin family at the cytoplasmic end of TM3 (Figure 1). Moreover, RhoprCCAPR has a conserved (NSxxNPxxY) motif element in the 7^th^ transmembrane region at amino acid residue 313 to 321 (Figures 1, 2A) [17].

#### Phylogenetic tree

*RhoprCCAPR* produces only one transcript (Figure 1) as has been observed in several species such as in Diptera (*C. quinquefasciatu, D. melanogaster, D. virile, D. mojavensis*), Hymenoptera (*A. florea, A. mellifera, B. impatiens, M. rotundata, N. vitripennis*) and Hemiptera (*A. pisum*); however, two isoforms of the CCAP receptor have been identified in three species of mosquito (*A. gambiae, Aedes aegypti, Culex pipiens*) and in *T. castaneum* and *B. mori* (Figure 2A, B). Phylogenetic analysis revealed that RhoprCCAPR belongs to the orthologous group of CCAP receptors in Hymenoptera including *A. florae, A. mellifera, B. impatiens, M. rotundata* and *N. vitripennis* (Figure 2B). RhoprCCAPR has high amino acid sequence similarity to identified or predicted CCAP receptors in Diptera, Coleoptera, Hymenoptera, Hemiptera, and Lepidoptera, with 55.8% pairwise identity (Figure 2A).

#### Spatial expression profile of the *RhoprCCAPR* gene

**Figure 3:**
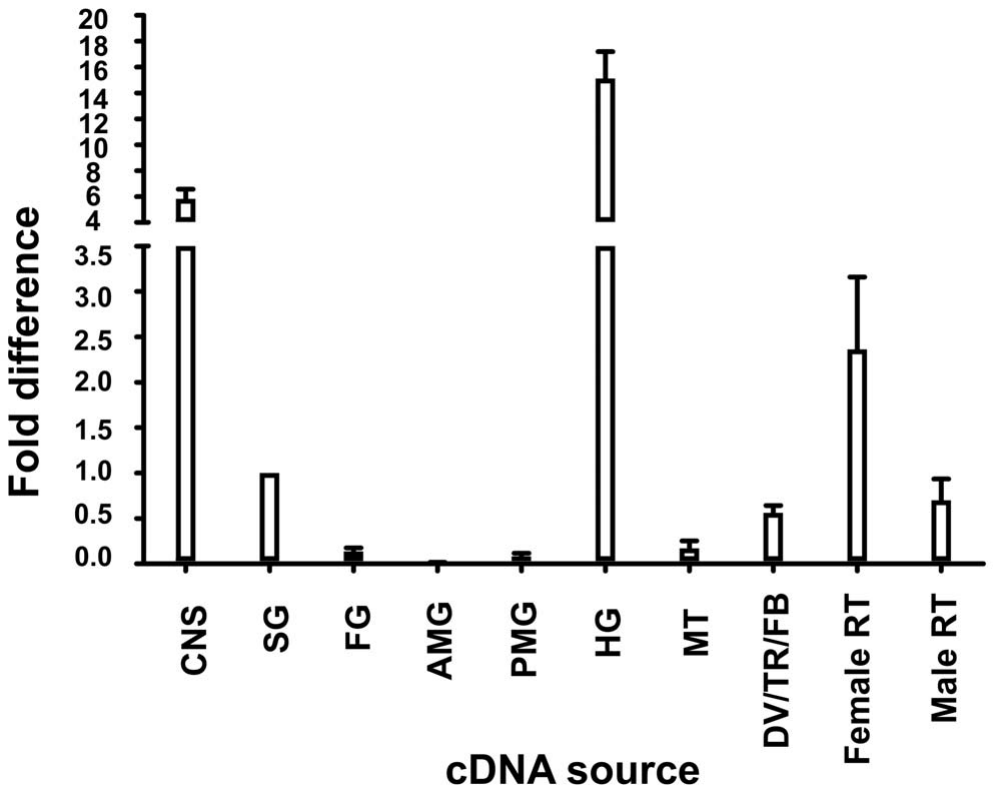
Expression profile of the *RhoprCCAP* receptor gene in fifth-instar *R. prolixus* tissues. A) *RhoprCCAPR* transcripts were observed in CNS as well as peripheral tissues. Fold-difference in expression is relative to *RhoprCCAPR* expression in the salivary glands. Abbreviations: CNS, central nervous system; SG, salivary glands; FG, foregut; AMG, Anterior midgut; PMG, Posterior midgut; HG, hindgut; MTs, Malpighian tubules; DV/TR/FB, dorsal vessel/trachea/fat body; Female RT, female reproductive tissue; Male RT, male reproductive tissue.

To identify the potential target sites of RhoprCCAP, the expression patterns of the putative *RhoprCCAPR* transcript were determined by real-time PCR (qPCR). The *RhoprCCAPR* gene expression was observed in the CNS, hindgut and female reproductive system (Figure 3). Also, lower transcript levels were observed in the 5^th^ instars salivary glands, the pool of tissues including dorsal vessel/trachea/fat body, and in the male reproductive tissues (Figure 3). On the other hand, very low or nearly undetectable levels of the transcript were observed in foregut, anterior midgut, posterior midgut, and Malphigian tubules (Figure 3).

#### Developmental expression profile of the *RhoprCCAPR* gene

**Figure 4:**
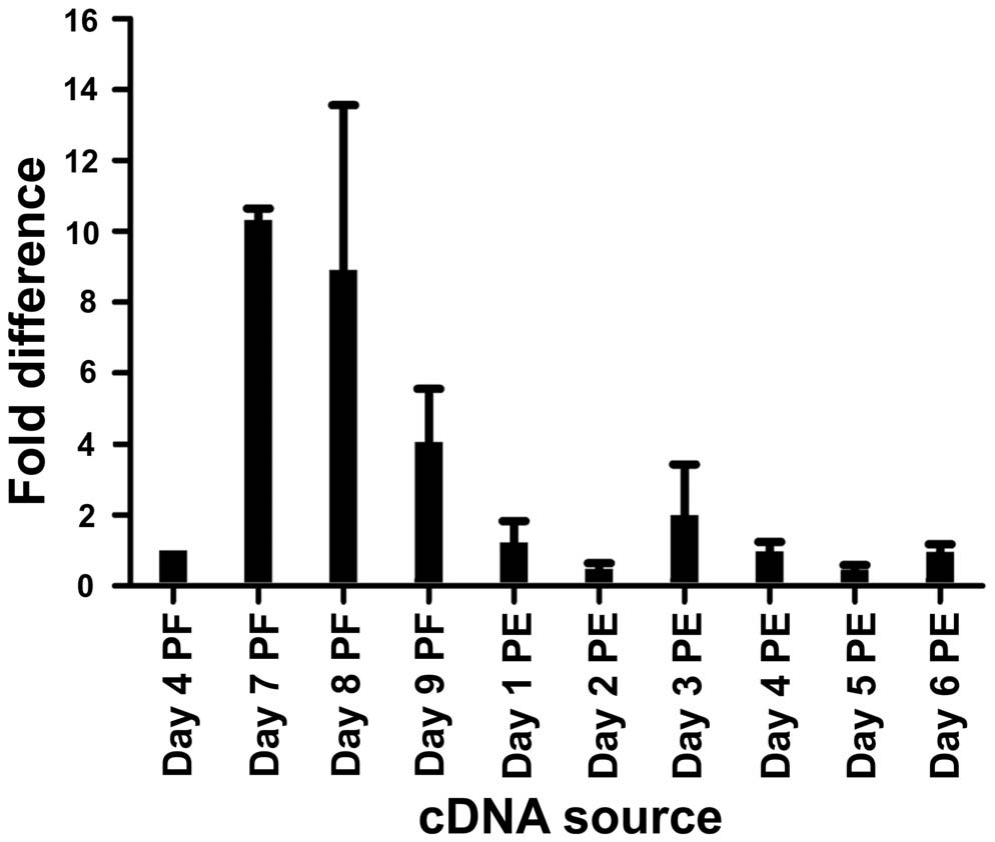
Expression profile of the *RhoprCCAP* receptor gene during development in a pool of tissues containing dorsal vessel/fat body/trachea of 4^th^ and 5^th^ instars. *RhoprCCAPR* expression level was increased prior to ecdysis and decreased post-ecdysis. Specifically, the peak *RhoprCCAPR* transcript level was observed in Day 7 post-fed (PF). Fold-difference in expression is shown relative to the expression of the *RhoprCCAPR* transcript in day 4 PF. Abbreviations: PF, post-fed; PE, post-ecdysis.

We were specifically interested in the developmental expression patterns of the putative *RhoprCCAPR* in the pool of tissues including dorsal vessel, trachea and fat body since CCAP is known to be a cardioacceleratory peptide. Our preliminary results showed that *RhoprCCAPR* transcript levels in day 2, 3 and 4 post-fed were not different. Since increasing heartbeat frequency right before ecdysis is essential in *D. melanogaster*, we chose day 4 post-fed as the earliest control. The *RhoprCCAPR* transcript level increased prior to ecdysis in 4^th^ instars, which is at 7 to 9 days post-feeding, and decreased post-ecdysis (Figure 4).

#### Functional receptor assays of the *RhoprCCAPR*

**Figure 5:**
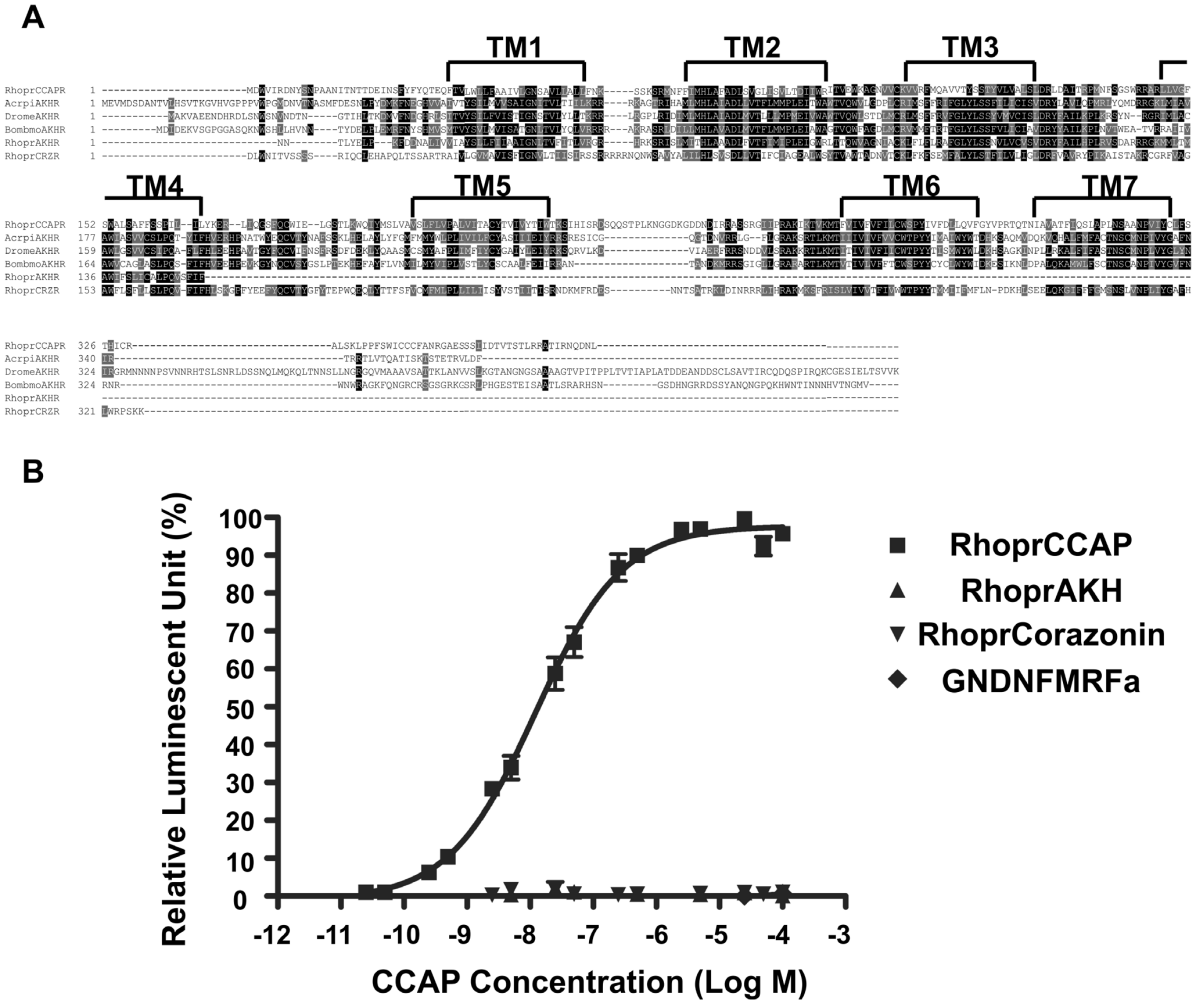
The alignments analysis of putative adipokinetic hormone (AKH) receptors, corazonin receptors and RhoprCCAP receptors as well as the RhoprCCAP receptor expression assay in (CHO-K1) WTA11 cells. A) Amino acid sequences of receptors for AKH and corazonin were compared to RhoprCCAPR. The RhoprCCAP receptor was aligned with the putative AKH and corazonin receptors in insects. Dark gray shading denotes sequences identical in greater than 50% of that particular column while light gray shading denotes similar residue to column-consensus residue. B) Activity of CCAP, AKH, corazonin and an extended FMRFamide in the RhoprCCAPR functional assay. Dose response curve shows the activity of CCAP on the expressed *RhoprCCAPR* has an EC_50_ of 12.25±1.1 nM.

To determine the endogenous ligand for the isolated putative RhoprCCAPR in *R. prolixus*, we used a calcium mobilization assay which expresses the *RhoprCCAPR* clone in CHO-K1 cells. Interestingly, the EC_50_ using CHO-K1 cells was quite high (341±8.8 nM). However, in CHO - WTA11 cells, RhoprCCAPR was dose-dependently activated by CCAP with an EC_50_ of 12.2±1.1 nM (Figure 5B). The receptor was not activated by other peptides that were tested, including RhoprAKH, RhoprCorazonin and an extended RhoprFMRFa, GNDNFMRFa (Figure 5A, 5B, Table 2). AKH and corazonin were chosen because receptor sequence alignments revealed that the receptors for these peptides may be structurally-related to the CCAP receptor. Control cells that were transfected with an empty pcDNA vector showed no response to the peptides that were used in our assay (data is not shown here). Thus, our control data illustrate that the functional receptor assay system is only activated in cells that are transfected with *RhoprCCAPR* and not by any endogenous receptors in these CHO cells.

#### CCAP function in heart rate

**Figure 6:**
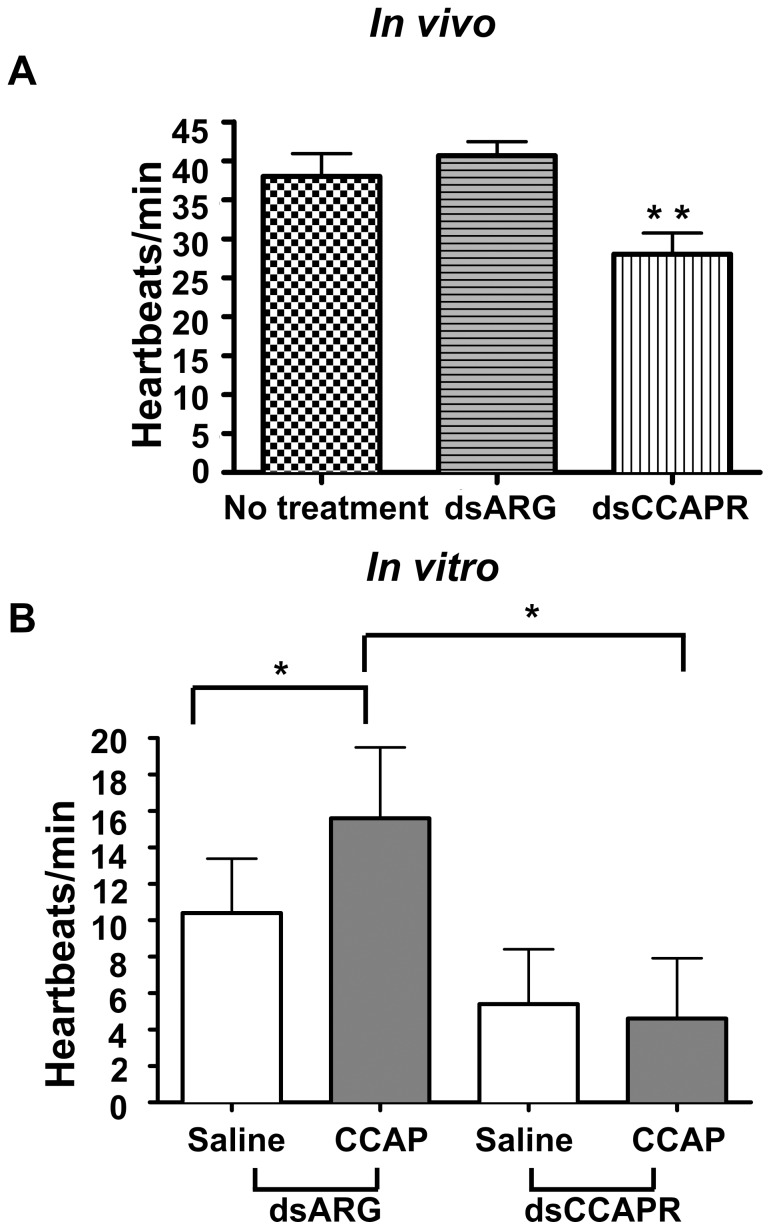
The effects of CCAP on heartbeat in adult male *R. prolixus*. 2 µg of dsRNA for the ampicillin resistance gene (dsARG) or for the RhoprCCAPR (dsCCAPR) was injected into adult male *R. prolixus* and 2 days after injection or no treatment heart rate was measured *in vivo* and *in vitro*. Values are expressed as the mean ± SEM (n = 5). Asterisk indicates significant differences between dsARG and *dsCCAPR*-treated groups of animals as determined by t-test (*P<0.05; **P<0.01). A) Heartbeat frequency of adult male *R. prolixus* was measured *in vivo* and was significantly lower in bugs in which *RhoprCCAPR* was knocked down. B) Heartbeat frequency was measured *in vitro* and found to be lower in *dsCCAPR*-treated bugs than those treated with dsARF. RhoprCCAPR knocked down bugs were also unresponsive to CCAP. White bars indicate heartbeats in saline and grey bars denote heartbeats in 10 ^−9^ M CCAP.

The effect of CCAP on the heartbeat frequency was studied *in vivo* and *in vitro* in the adult male *R. prolixus* treated with control (dsRNA) or dsCCAPR. *In vivo* heartbeat frequency of dsCCAPR-treated bugs (28.0±2.7 beats/min, n = 10) was significantly decreased by 31.1% compared to the dsARG treated group (40.7±1.8 beats/min, n = 10) (paired t test, p = 0.0005) (Figure 6A). Previously, we have shown that CCAP increases heartbeat frequency *in vitro* in 5^th^ instar *R. prolixus* [33]. To verify the results observed *in vivo*, we investigated whether the reduced heartbeat frequency was due to the absence of the CCAPR. Thus, we again knocked down the *RhoprCCAPR* mRNA and measured heartbeat frequency *in vitro*. Our results showed that the heartbeat frequency of the dsARG treated bugs was 10.4±2.9 beats/min in saline and was significantly increased to 15.6±3.9 beats/min in the presence of 10^−9 ^M CCAP (paired t test, p = 0.0376) (Figure 6B). In contrast, the heartbeat frequency of the group that was treated with dsCCAPR was 5.4±3.0 beats/min in saline and was 4.6±3.3 beats/min, in the presence of 10^−9^M CCAP (paired t test, p = 0.1688) (Figure 6B). When we compared the heartbeat frequency of the two groups in saline that were treated with dsARG or dsCCAPR, the difference was lower, but not statistically significant (unpaired t test, p = 0.1357). However, when the results of after CCAP application was compared between the two groups, the difference was statistically significant (unpaired t test, p = 0.0318) (Figure 6B). The percentage knock-down of the *RhoprCCAPR* transcription was quantified by qPCR in the pool of tissues (dorsal vessel/trachea/fat body) from these insects and was found to be knocked down by 80.3±1.5% 2 days after injection relative to control dsARG injected bug.

## Discussion

The GPCR superfamily is a critical target for developing pharmacological drug treatments and more than one third of current human drugs act on this family [1], . Identifying agonists or antagonists for this receptor family can lead to treatment for many human diseases, or the development of novel pest-control agents [5], [6], [30].

The CCAP receptor in insects belongs to the GPCR superfamily and it has been characterized in Diptera, Coleoptera, Hymenoptera, Hemiptera and Lepidoptera [19]–[22]. In the present study, we have isolated and characterized the CCAP receptor from *R. prolixus.* The RhoprCCAPR shows high amino acid sequence similarity to CCAP receptors identified in Hymenoptera, including *A. mellifera*, *A. florea*, *B. impatiens*, *M. rotundata* and *N. vitripennis*. Interestingly, two isoforms of the CCAP receptor have been isolated in some Diptera (*A. gambiae, A. aegypti, C. pipiens*), Coleoptera (*T. castaneum*) and Lepidoptera (*B. mori*) but not in other Diptera (*Drosophila*), Hymenoptera or Hemiptera.

We showed the spatial expression patterns of the *CCAPR* in *R. prolixus* and this correlates with our previous immunohistochemical data and physiological roles of CCAP [31]. We have previously shown cells and processes containing CCAP-like immunoreactivity are distributed throughout the CNS and associated with the heart in *R. prolixus* [31] and CCAP increases heartbeat frequency and contraction in a dose-dependent manner in the heart and hindgut, respectively [31]. Thus the expression of *RhoprCCAPR* in the CNS and peripheral tissues confirms these findings, suggesting that CCAP controls central and peripheral physiological processes. Expression of *CCAPR* transcripts has been investigated in *D. melanogaster* [19], *T. castaneum* [32], [36] and *A. gambiae* [22]. Its expression was observed in all developmental stages from embryonic stages to adult in *D. melanogaster, A. gambiae* and *T. castaneum*. Specifically, the peak *CCAPR* expression was observed during late pupal stages in *D. melanogaster* and *A. gambiae* while it was observed in the early adult of *T. castaneum* [19], [21], [22]. The transcript was mainly observed in the head in adult fly and mosquito [19], [22].

Interestingly, we observed additional tissues that expressed the *CCAPR,* such as the salivary glands, and female and male reproductive systems. This suggests additional targets for the endogenous CCAP. The *R. prolixus* salivary gland has a double layer of visceral muscle surrounding a large secretory cavity and these muscles are under the control of various neuropeptides and serotonin [32]. Thus, CCAP might also be involved in the control of muscle contraction of the salivary glands or in the process of salivary secretion. Also, the presence of the RhoprCCAPR in the reproductive systems of male and female *R. prolixus* indicates that CCAP may be involved in reproduction, as it has been shown in *L. migratoria* [14]. Future studies are required to investigate the other physiological roles of endogenous CCAP at these newly identified target tissues.

To support our phylogenetics and alignment analysis with the expression profile, which suggests that the identified receptor was a CCAP receptor homolog, we expressed this receptor in CHO cells. The expressed *CCAP* receptor was only activated by low concentrations of RhoprCCAP with EC_50_ = 12.2±1.1 nM when tested in CHO-WTA11 cells. The improved sensitivity of this cell line over CHO-K cells is due to presence of the promiscuous Gα proteins. Interestingly, although the putative CCAP receptor has been isolated in 20 species of arthropods, the CCAP receptor has only been deorphanized in three species including *D. melanogaster*, *A. gambiae* and *T. castaneum*. In *D. melanogaster*, the CCAP receptor (CG6111) is activated by low concentrations of CCAP (EC_50_ of 5.4 x10^−10^M) [19]. In *A. gambiae*, the CCAP receptor is activated at an EC_50_ of 1nM CCAP [20] and in *T. castaneum*, two CCAP receptors, CCAPR1 and CCAPR2, are activated by CCAP with an EC_50_ of 624nM and 22nM respectively [21]. The difference in EC_50_ values may be due to techniques and the expression system that was used, but our EC_50_ value of 12.2nM is comparable to that found in *D. melanogaster* and *A. gambiae*.

We also investigated whether RhoprCCAPR can be activated by other peptides, including corazonin and AKH because the CCAP receptor alignment analysis with corazonin and AKH receptors reveals that they may be structurally-related (31.2% pairwise identity). Also, these peptides are functionally inter-related. For example, CCAP influences AKH release from the corpora cardiaca in *Schistocerca gregaria* [38] [33]. In *M*. *sexta*, AKH mobilize lipids from the fat body during flight or locomotion when heartbeat frequency also increases. Also, CCAP, AKH and corazonin increase heartbeat frequency in some insects [39] [34]. However, this possible functional inter-relationship does not extend to the agonist-binding properties of the corresponding GPCRs, since neither AKH nor corazonin activate the RhoprCCAPR, and nor does an unrelated peptide, an extended FMRFamide.

The crucial roles of CCAP in regulating ecdysis behaviour have been studied in *D. melanogaster*, *Manduca sexta* and *T. castaneum* [36], [40]–[42]. In the moth, assays using the isolated abdominal CNS suggest that CCAP is required for turning off the pre-ecdysis motor program [40] and turning on the ecdysis motor behaviours [40.41]. In *Drosophila*, lack of CCAP neurons results in the complete failure of pupal ecdysis [42]. Arakane *et al* (2008) showed that when transcripts levels of *CCAP* and its *CCAP* receptor were reduced, ecdysis behaviors were interrupted in *T. castaneum* [36]. Interestingly, in *D. melanogaster*, Baker et al. (1999) showed that the *Drosophila* heartbeat frequency was increased during the last 10 h of adult development and peaked at 1 hour before ecdysis at the white stage [43]. If CCAP has a role in increasing heartbeat frequency prior to ecdysis, then we should expect that the *CCAP* receptor expression might be up regulated at this time. As anticipated, the *CCAP* receptor mRNA levels in the pool of tissues (dorsal vessel/trachea/fat body) were increased up to 10 fold prior to ecdysis and decreased post-ecdysis. In addition, CCAP receptor expression was high in the hindgut, a tissue that is regulated at ecdysis for gut emptying and elimination of its cuticular lining. Our results certainly suggest that CCAP might play important roles in *R. prolixus* ecdysis and future studies will examine this.

The involvement of CCAP in cardiac function has been studied in several insects, including its involvement in adult wing inflation in *M. sexta* (see 8) In *D. melanogaster*, although CCAP RNAi injection was not found to have any effect on heartbeat frequency, CCAP cell ablation resulted in a heart rate that was decreased by 37–51%. CCAP cell ablation only affected the anterograde phase of the heartbeat suggesting that CCAP may be involved in regulating the anterograde pacemaker in *D. melanogaster*. In *A. gambiae*, silencing the CCAP transcript resulted in a statically significant (6% and 7%) reduction in the total and anterograde heart rate [22]. Previously, we demonstrated that CCAP increases the frequency of the heartbeat in *R. prolixus* in a reversible, dose-dependent manner [33] and reducing endogenous CCAP receptor levels by RNAi lowers heartbeat *in *vivo and eliminates the CCAP-induced increase *in vitro.* The basal heart beat rate *in vivo* is higher than that observed *in vitro*, which might imply the absence of endogenous cardioaccelators in the *in vitro* condition. This is the first report to show that reducing CCAP receptor transcript levels leads to a reduction in the cardiac output in any insect. Interestingly, heartbeat rate is reduced in dsCCAPR-treated bugs indicating that normal heartbeat rate is elevated due to the presence of endogenous CCAP.

The very low or nearly undetectable levels of receptor transcript observed in anterior midgut and posterior midgut in *R. prolixus* is of some interest, since in *P. americana* CCAP up-regulates the activity of digestive enzymes in midgut [10], [44]. Thus, exposure of isolated midgut to CCAP increases α-amylase and protease activity. The CCAP may act in a paracrine manner, released from CCAP-containing midgut endocrine cells [10], [44]. Clearly *R. prolixus* appears to be different, since there does not appear to be CCAP receptors associated with the midgut, and previous studies have failed to find CCAP-like immunoreactive endocrine cells in *R. prolixus* midgut [33].

*R. prolixus* is the principal vector of Chagas’ disease, and transmits the parasitic protozoan, *Trypanosoma cruzi*. Currently, the best solution for disrupting the transmission of this disease is by controlling the vector. Hence, *R. prolixus* is a useful model organism for studying physiological and neuroendocrine processes but is also medically important. Since GPCRs act as key regulators in the physiology of insects (and other animals) and are critical drug targets in human medicine and in the agricultural industry, understanding and characterizing GPCRs in this insect could lead to control measures against the transmission of Chagas’ disease.
